# Successful endoscopic closure with an over-the-scope clip for sigmoid colon perforation due to bile duct stent migration

**DOI:** 10.1007/s12328-021-01544-x

**Published:** 2021-10-29

**Authors:** Daisuke Yamaguchi, Goshi Nagatsuma, Azuki Jinnouchi, Yumi Hara, Akane Shimakura, Amane Jubashi, Wataru Yoshioka, Yuichiro Tanaka, Naoyuki Hino, Keisuke Ario, Seiji Tsunada

**Affiliations:** 1 Department of Gastroenterology, National Hospital Organization Ureshino Medical Center, Ureshino, Japan; 2 Division of Gastroenterology, Department of Internal Medicine, Saga University, Saga, 849-8501 Japan

**Keywords:** Over-the-scope clip, Endoscopic closure, Bile duct stent, Stent migration, Stent perforation

## Abstract

An 86-year-old woman presented with a history of endoscopic papillary sphincterotomy for bile duct stones and diverticulitis. The patient was admitted as an emergency case of acute cholangitis due to choledocholithiasis, underwent endoscopic bile duct stenting, and was discharged with a plan for endoscopic lithotripsy. One month later, the patient was readmitted owing to abdominal pain. Abdominal computed tomography at admission showed that the bile duct stent had migrated to the sigmoid colon and the presence of a small amount of extraintestinal gas, suggesting a colonic perforation. Lower gastrointestinal endoscopy showed adhesions and intestinal stenosis in the sigmoid colon, probably after diverticulitis, and the bile duct stent that had perforated the same site. The stent was removed and endoscopic closure of the perforation was performed using an over-the-scope clip. Abdominal computed tomography 8 days after the closure showed no extraintestinal gas. The patient resumed eating and was discharged on the 14th day of admission. There was no recurrence of abdominal pain. Endoscopic closure of sigmoid colon perforation due to bile duct stent migration using an over-the-scope clip has not been reported thus far, and it may be a new treatment option in the future.

## Introduction

Endoscopic retrograde cholangiopancreatography (ERCP) plays an important role in the diagnosis and treatment of biliary and pancreatic diseases [1]. Endoscopic bile duct stenting in ERCP has been widely performed in recent years for biliary obstruction. Migration of a biliary stent is a known potential complication of ERCP, with distal migration occurring in 4%–6% cases [2, 3]. However, gastrointestinal penetration or transmural perforation due to stent migration is rare, with an incidence of < 1% [4]. Perforation due to displacement of the biliary stent can occur mainly in the duodenum and in other parts of the small intestine and the colon [5–9]. In recent years, endoscopic closure of gastrointestinal perforations using an over-the-scope clip (OTSC) has been shown to be effective with a high success rate [6, 10–14]. Here, we have reported a case of sigmoid colon perforation caused by a bile duct stent that was treated conservatively by endoscopic closure using an OTSC.

## Case report

**Fig. 1:**
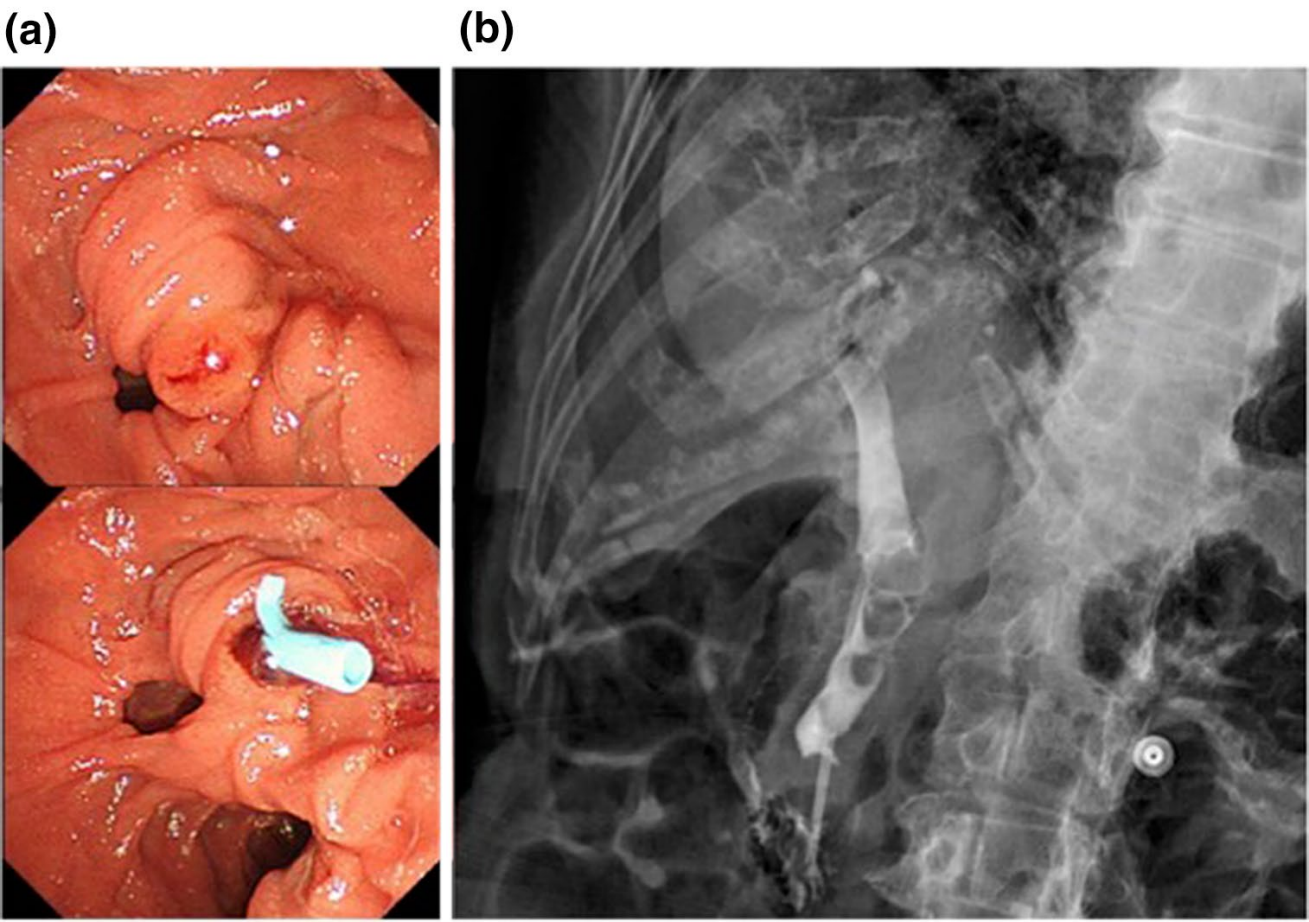
Bile duct stent placement during ERCP for acute cholangitis **a** Endoscopic image **b** Fluoroscopic image

An 86-year-old woman was admitted to our hospital due to fever and abdominal pain. Blood test results showed elevated inflammatory markers and liver function test parameters. Abdominal computed tomography (CT) showed dilated bile ducts and stones in the common bile duct. Since the patient was diagnosed with acute cholangitis, ERCP (on the same day) and endoscopic bile duct stenting were performed (7 Fr × 7 cm straight plastic stent [QuickPlace V; Olympus, Japan]; Fig. 1). After the interventions, cholangitis improved, and the patient was discharged with a plan to undergo elective endoscopic lithotripsy in two months.

The patient had a history of endoscopic papillary sphincterotomy for bile duct stones and several episodes of diverticulitis of the sigmoid colon.

**Fig. 2:**
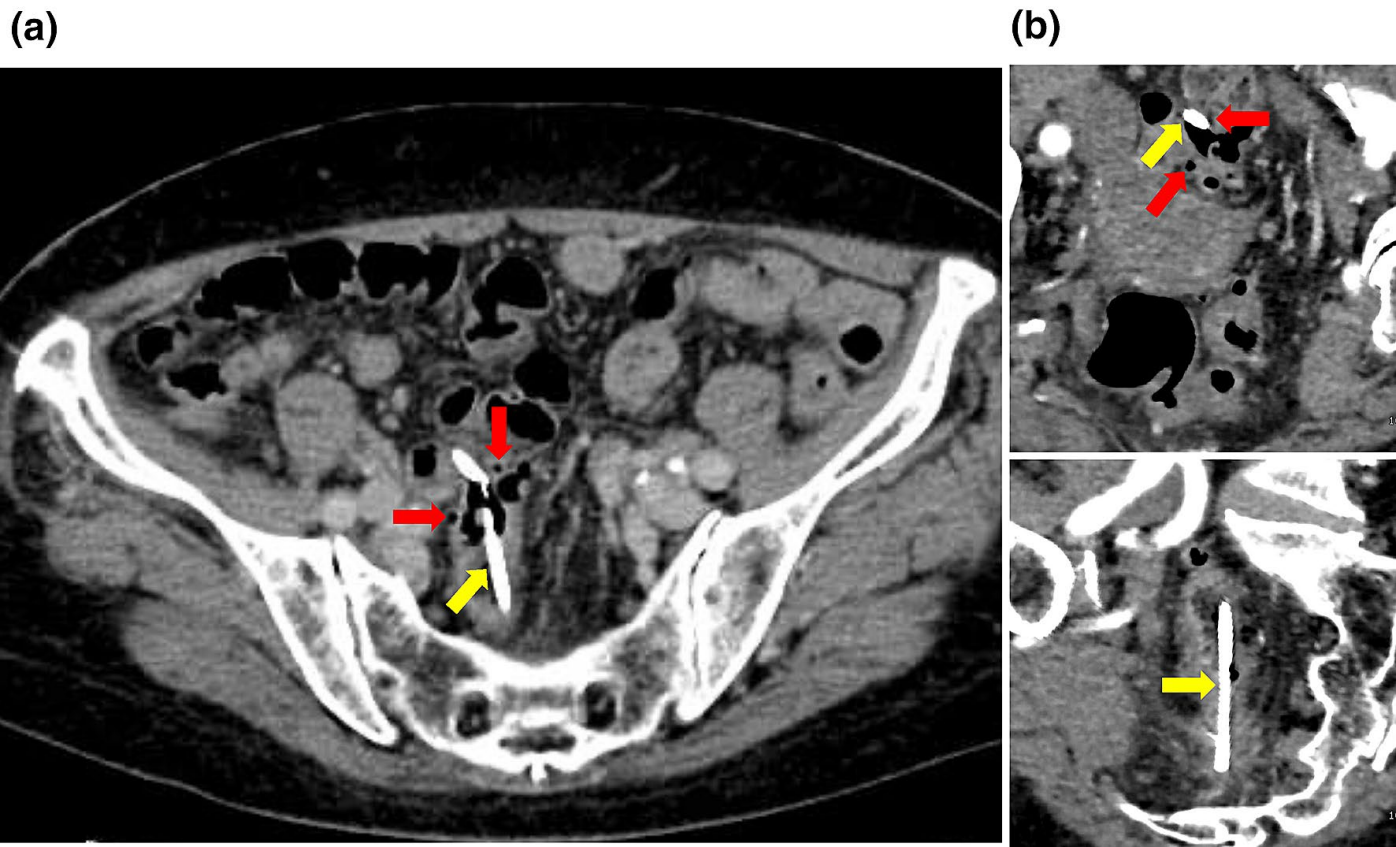
CT image of the abdomen on admission **a** Sigmoid colon perforation (yellow arrow) and the extraintestinal gas (red arrow) **b** Tip of the stent perforating the intestine (yellow arrow) and the extraintestinal gas (red arrow)

**Fig. 3:**
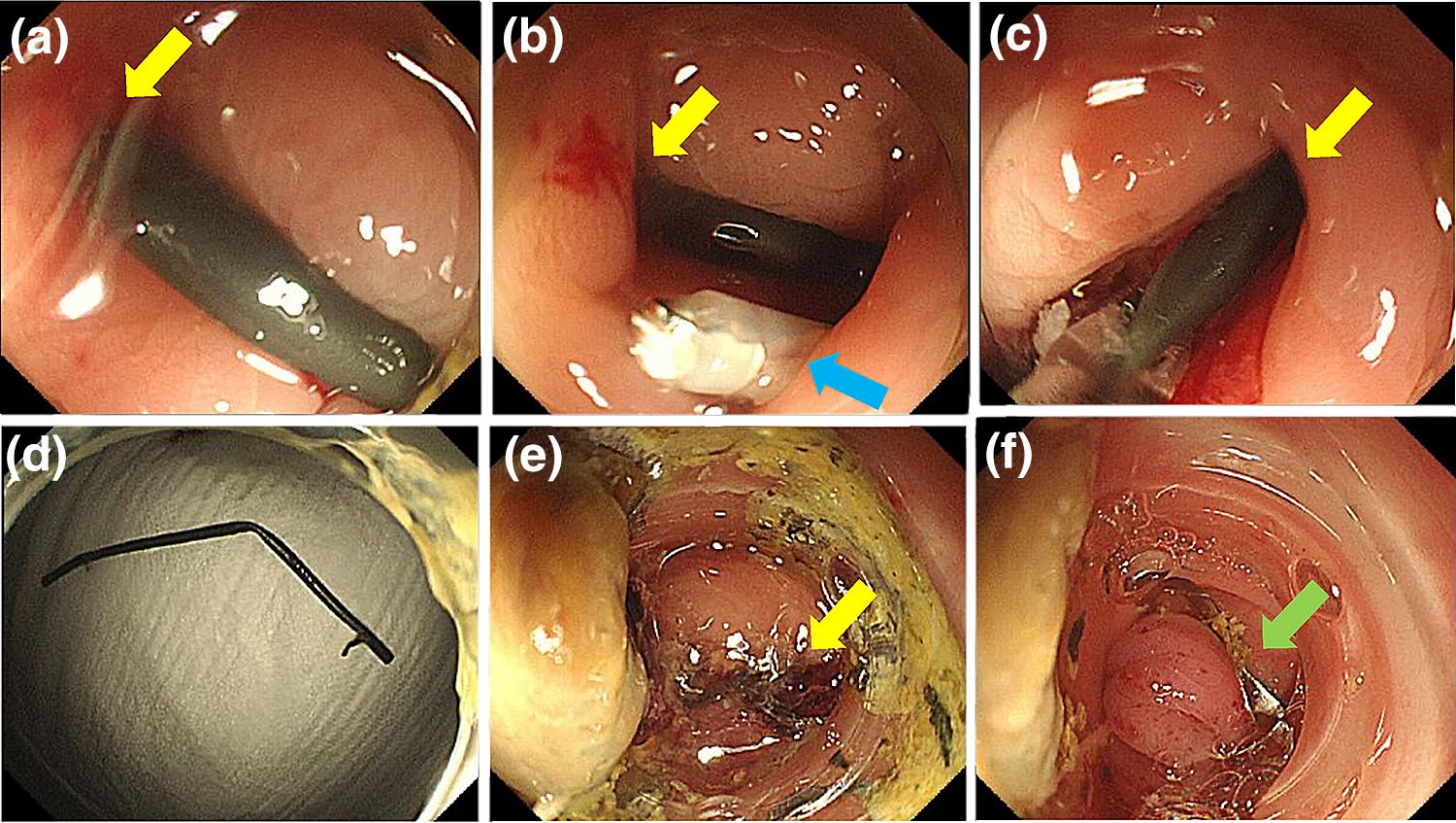
Endoscopic OTSC closure **a** Sigmoid colon perforation (yellow arrow) **b** Hit the marking clip (blue arrow) **c** Removal of the stent with grasping forceps **d** Removed stent **e** OTSC placement and confirmation of perforation site (yellow arrow) **f** End of closure with an OTSC (green arrow)

**Fig. 4:**
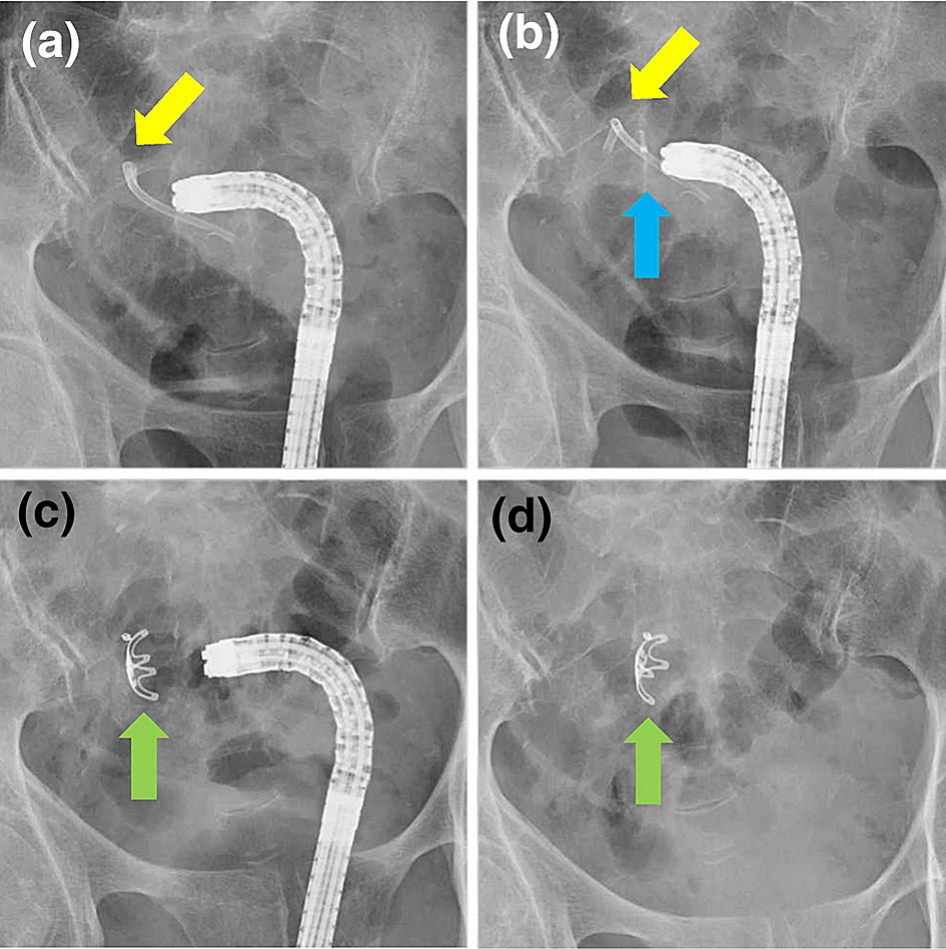
Fluoroscopic images during OTSC closure **a** Sigmoid colon perforation (yellow arrow) **b** Hit the marking clip (blue arrow) **c** OTSC placement (green arrow) **d** End of closure with the OTSC (green arrow)

**Fig. 5:**
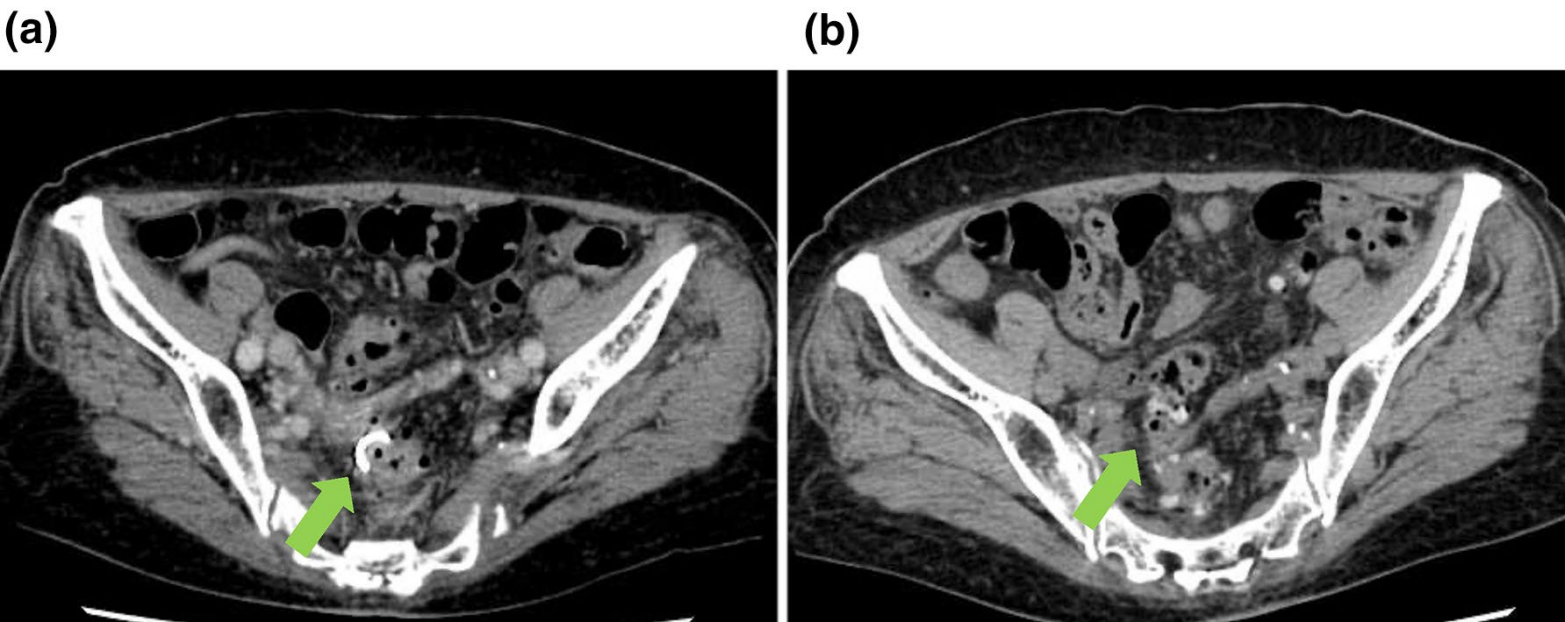
CT image of the abdomen **a** On the eighth day of hospitalization, with remains of the OTSC (green arrow) and no extraintestinal gas **b** One month after discharge, spontaneous detachment of the OTSC (green arrow)

A month after discharge, the patient was readmitted to our hospital due to abdominal pain. Blood test results showed elevated inflammatory markers, but no elevation in liver function test parameters. Abdominal CT at admission showed that the bile duct stent had migrated to the sigmoid colon (Fig. 2a). The tip of the stent protruded through the wall of the sigmoid colon, and there was a small amount of extraintestinal gas around the stent, suggesting sigmoid colon perforation by the stent (Fig. 2b). Although we considered emergency surgery, we decided to treat the patient conservatively with fasting and antimicrobial administration because the patient’s vitals were stable and the patient had minimal extraintestinal gas, mild pain, and no abdominal muscular defenses. Lower gastrointestinal endoscopy performed a day after admission showed intestinal adhesions and stenosis in the sigmoid colon, probably due to diverticulitis, and the bile duct stent had perforated the same site (Fig. 3a, Fig. 4a). Endoscopic manipulation was difficult due to the stenosis of the sigmoid colon, and a marking clip was first placed near the perforation site (Fig. 3b, Fig. 4b). Next, the stent was removed by endoscopy using grasping forceps (Fig. 3c, d, Fig. 4b). An OTSC (12 × 6 mm GC; Ovesco Endoscopy AG, Germany) was then placed, the endoscope was reinserted, the perforation was confirmed (Fig. 3d), and the site of perforation was closed using the OTSC (Fig. 3e, f, Fig. 4c, d). Conservative treatment with fasting and antimicrobial agents was continued. On the eighth day of admission, abdominal CT showed no extraintestinal gas (Fig. 5a) and reduction in the inflammatory reaction; therefore, the patient was allowed to resume eating. After the resumption of meals, the patient did not have a fever or abdominal pain and was discharged on the 14th day of admission.

After discharge from the hospital, the patient did not experience abdominal pain, and 1 month after discharge, abdominal CT showed spontaneous detachment of the OTSC (Fig. 5b). Further, endoscopic lithotripsy was performed for the common bile duct stones, and the patient has been doing well since then.

## Discussion

Endoscopic bile duct stenting is frequently used for the treatment of cholangitis, and stent migration is often observed as an incidental complication. The complication rate of biliary stents is 8–10%, and the most common complication is the migration of the proximal or distal part of the stent [2]. Duodenal perforation secondary to distal stent migration has been well documented in the literature. However, due to the low incidence of this life-threatening complication (< 1%) [4], prevention and treatment methods are controversial.

In our case, the patient had undergone endoscopic papillary sphincterotomy before the occurrence of bile duct stent migration. Endoscopic sphincterotomy (EST) before stent placement and a long biliary stent are considered risk factors for migration of the distal part of the stent rather than the proximal part of the sent [2]. In addition to these risk factors, Arhan et al. [15] have reported that biliary stent migration is more likely to occur in cases of benign biliary stricture than in cases of malignant biliary stricture. Several reports have also shown that all migrated biliary stents are of the straight type and are more likely to migrate distally than pigtailed biliary stents [5, 16]. The double pigtail biliary stent may have an anti-migration effect as it is fixed in the bile duct, and its flexible and soft plastic may prevent intestinal perforation due to its migration. The use of pigtail stents should be considered in post-EST cases, as in this case.

Bile duct stents are rarely associated with colonic perforations. Miyasaka et al. [17] reported 22 cases of colonic perforation due to bile duct stent migration in Japan, five of which were treated with endoscopic treatment by clipping. Among cases that underwent surgery, partial resection, wedge resection, and suture closure were performed in eight cases, Hartmann’s operation was performed in five cases, and suture closure and colostomy were performed in two cases. The stent perforations were small in area and the stent lumens were narrow, which prevented feces from flowing out of the intestine; therefore, the treatment was relatively minimally invasive for colonic perforations. The sigmoid colon is often reported as a site of colorectal perforation [18, 19]. In our case, the sigmoid colon was stenotic due to repeated sigmoid diverticulitis, and the migrated bile duct stent was thought to have lodged in the sigmoid colon, resulting in a perforation.

An OTSC can close all layers of the gastrointestinal wall [20], and its efficacy has been reported in cases of gastrointestinal perforation, fistula, and refractory bleeding after endoscopic treatment [10]. Haito-Chavez et al. [21] reported suture of 14 colorectal perforations using an OTSC. Voermans et al. [22] used an OTSC in 13 cases of medically induced colorectal perforation caused by endoscopy, polypectomy, or percutaneous drainage and reported a clinical improvement rate of 92% (12/13 cases).

As shown in Table 1, 13 cases, including our case, of endoscopic closure using an OTSC for gastrointestinal perforation due to bile duct stent migration have been reported thus far [6, 12–15]. Endoscopic closure was successful in all 13 cases, and OTSC closure was considered safer than clip closure. However, two deaths were reported during the postoperative period. Therefore, the patient’s condition should be monitored after OTSC closure, and surgical intervention should be considered if necessary. To our knowledge, our case is the first case of OTSC closure for sigmoid colon perforation. In our case, there was no peritonitis or abscess formation around the sigmoid colon perforation, and after endoscopic removal of the bile duct stent, the perforation was closed using an OTSC, which allowed conservative treatment. In addition, the sigmoid colon was stenotic due to repeated diverticulitis, making endoscopic manipulation difficult and clipping at the targeted location impossible. Even when clipping was difficult, OTSC was able to close the lesion if it could get close enough. OTSC closure is effective for perforation due to bile duct stents with a small perforation area.

**Table 1:** OTSC closure for perforation due to plastic biliary stent migration

Author	Age	Sex	Diagnosis	Stent type	Symptoms	Perforatio*n* site	Time to stent migration (Days Post-ERCP)	Outcome of the Endoscopic procedure	Overall outcome of the patient
Kriss M, et al. [13]	48	Male	Hilar biliary stricture after liver transplantation	Unmentioned size, straight	Fever and abdominal pain	Duodenum	14	Successful with OTSC	Improved
Le Mouel JP, et al. [14]	71	Male	Cholangiocarcinoma	12 cm 8.5 Fr, straight	Fever and abdominal pain	Duodenum	1	Successful with OTSC	Improved
Bureau MA, et al. [11]	75	Male	Biliary leak post hepatectomy	18 cm 8.5 Fr, double flaps	Fever and abdominal pain	Duodenum	4	Successful with OTSC	No further interventions were required, or complications were observed 28 days later
Bureau MA, et al. [11]	61	Male	Ischemic cholangiopathy	7 cm 8.5 Fr, sigmoid shaped	Fever and abdominal pain	Duodenum	2	Successful with OTSC	No further interventions were required, but the patient died 17 days later (from biliary sepsis)
Bureau MA, et al. [11]	31	Female	Choledocholithiasis and bile duct stenosis	15 cm 7 Fr, double flaps	Fever and abdominal pain	Duodenum	4	Successful with OTSC	No further interventions were required, or complications were observed 28 days later
Bureau MA, et al. [11]	52	Male	Ischemic cholangiopathy	12 cm 8.5 Fr, double flaps	Fever and abdominal pain	Duodenum	90	Successful with OTSC	No further interventions were required, or complications were observed 28 days later
Bureau MA, et al. [11]	72	Male	Bile duct compression after hepatic artery embolization	13 cm 8.5 Fr, double flaps	Fever and abdominal pain	Duodenum	2	Successful with OTSC	Had peritonitis requiring laparotomy and died at day 5 post perforation
Bureau MA, et al. [11]	45	Female	Anastomotic stenosis post liver transplantation	12 cm 8.5 Fr, double flaps	Fever and abdominal pain	Duodenum	2	Successful with OTSC	No further interventions were required, or complications were observed 28 days later
Gromski MA, et al. [6]	56	Male	Metastatic cholangiocarcinoma	18 cm 10 Fr, plastic biliary stent in left intrahepatic duct	Right upper quadrant pain, fever	Duodenum	47	Successful with OTSC	Improved
Thapa N, et al. [12]	65	Female	Cholangiocarcinoma	12 cm 10 Fr, straight	Severe right upper quadrant pain	Duodenum	60	Successful with OTSC	Improved, Discharge 2 days later
Thapa N, et al. [12]	28	Male	Anastomotic stricture post liver transplant	12 cm 10 Fr, straight	Elevated liver function tests	Duodenum	30	Successful with OTSC	Improved, Discharge 2 days later
Our Case	86	Female	Choledocholithiasis	7 cm 7 Fr, straight	Abdominal pain	Sigmoid colon	30	Successful with OTSC	Improved, Discharge 14 days later

In conclusion, endoscopic closure with an OTSC may be a new treatment option for sigmoid colon perforation caused by bile duct stent migration.
